# Hemoglobin mass does not increase in able-bodied individuals after consecutive days of acute intermittent hypoxia

**DOI:** 10.3389/fphys.2025.1714165

**Published:** 2025-12-03

**Authors:** Andrew S. Harding, Katelyn D. Noyes, Aviva K. Pollet, Sewan Kim, Andrew Q. Tan

**Affiliations:** 1 NeuroRecovery Laboratory, Department of Integrative Physiology, University of Colorado Boulder, Boulder, CO, United States; 2 Applied Exercise Science Laboratory, Department of Integrative Physiology, University of Colorado Boulder, Boulder, CO, United States; 3 Center for Neuroscience, University of Colorado, Boulder, CO, United States; 4 Rocky Mountain Regional VA Medical Center, U.S. Department of Veterans Affairs, Aurora, CO, United States

**Keywords:** Erythropoietin, oxygen saturation, cardiorespiratory fitness, altitude, hemoglobin mass

## Abstract

Prolonged exposure to hypoxia can elevate serum erythropoietin (EPO) and may eventually increase hemoglobin mass. However, whether intermittent hypoxia consisting of breathing short intervals alternated with normoxia (acute intermittent hypoxia; AIH) can trigger erythropoiesis and lead to increases in hemoglobin mass is less clear. Therefore, the purpose of this study was to investigate the effect of consecutive days of AIH on hemoglobin mass. Participants (n = 18) were exposed to 4 consecutive days of AIH consisting of fifteen 90 s intervals of breathing ∼9% O_2_ alternating with 60 s of breathing room air (∼21% O_2_). Hemoglobin mass was measured in each participant before the first and after the last exposure. In a separate group of individuals (n = 12) we collected serum blood samples for EPO analysis before and 4.5 h after one of the AIH sessions, as well as at the same timeframe on a day with no AIH to serve as control. There was no significant increase in hemoglobin mass after four consecutive days of AIH in the first group with no serum EPO collection, or in the second group for which we collected EPO. Further, there was no significant increase in serum EPO after AIH as compared to control. These results indicate that four consecutive days of AIH is not a sufficient hypoxia exposure to elicit increases in hemoglobin mass in able-bodied individuals. Serum EPO results suggest that a single session of the current acute intermittent hypoxia protocol does not provide enough stimulus for EPO production.

## 1 Introduction

Exposure to hypoxia can stimulate the production of erythropoietin (EPO), which promotes the proliferation and differentiation of erythrocytes (Jelkmann, 2011). When elevated serum EPO levels are sustained, red cell volume and hemoglobin mass have been shown to increase (Siebenmann et al., 2015). Additionally, intermittent exposures to altitude or hypoxia consisting of interval lengths that vary from hours to days have been explored as means of increasing hemoglobin mass (Schmidt, 2002). For example, 7 days of intermittent hypoxia consisting of 12 h of hypoxia alternated with 12 h of normoxia significantly increases plasma EPO and reticulocyte count (Koistinen et al., 2000). In addition, 6 months of alternating between 11 days at altitude and 3 days at sea level significantly increases hemoglobin mass (Heinicke et al., 2003) However, whether intermittent hypoxia consisting of much shorter intervals (<5 min) can increase serum EPO and, eventually, hemoglobin mass is unclear.

Studies of varying brief intermittent hypoxia protocols have shown the potential of short intermittent hypoxia to increase EPO and/or hemoglobin mass. Studies using one session of eight 4 min hypoxia exposures with 1–2 min breaks of breathing normoxia have shown increases in EPO but not hemoglobin mass in healthy adults and older individuals (Wojan et al., 2021; 2023). While one session of hypoxia did not increase hemoglobin mass, additional perspective is offered by studies that have implemented short duration intermittent hypoxia across consecutive days. For example, five consecutive days of 4–6 min intermittent hypoxia intervals with less than 2 minutes rest in between for approximately 70 min each day significantly increases red blood cell counts in young adults on the fifth day of hypoxia (Tobin et al., 2022). While an elevated red blood cell count offers important information, it does not reliably reflect underlying changes in hemoglobin mass.

Furthermore, consecutive days of an intermittent hypoxia protocol consisting of fifteen 90 s intervals with 60 s breaks (acute intermittent hypoxia; AIH) have been investigated due to its role in improving motor function in individuals with incomplete spinal cord injury (iSCI) (for a review see (Tan et al., 2020a). Emerging investigations have indicated that there may also be a cardiorespiratory benefit to AIH. For example, initial research in able-bodied individuals have suggested AIH improves the metabolic cost associated with walking during a motor learning task (Bogard et al., 2024; 2025). Additionally, AIH combined with walking training improves walking speed and endurance compared to walking training alone in individuals with iSCI (Tan et al., 2021). Given that hemoglobin mass and cardiorespiratory fitness are well-correlated (Schmidt and Prommer, 2010) and that AIH has the potential to increase hemoglobin mass, the contributions of possible changes in hemoglobin mass to walking improvements after AIH warrant investigation. Therefore, the purpose of this study was to identify whether hemoglobin mass could have increased during this AIH protocol, potentially explaining some of the documented walking improvements. Further identifying the mechanisms by which AIH may improve cardiorespiratory fitness in all individuals may improve the understanding of the benefits of this therapy in able-bodied individuals and individuals with iSCI alike.

## 2 Methods

### 2.1 Study design

The present investigation was embedded within a larger project examining the effects of AIH on corticospinal excitability and biomarkers of neuroplasticity (Pollet et al., In Review). Detailed descriptions of the overall study design and primary outcomes are reported elsewhere. The secondary outcomes presented in this study are novel and have not been published previously. In short, participants completed four consecutive days of AIH with hemoglobin mass measured before the first session of AIH and after the last session of AIH. In a separate protocol, participants were recruited to complete only the AIH and hemoglobin mass procedures, and venous blood for EPO analysis was collected before and 4.5 h after a control visit and the first AIH session. This study was approved by the Colorado Multiple Institutional Review Board (COMIRB #23-1275) and all participants provided written informed consent. All procedures conformed to the standards set by the *Declaration of Helsinki*.

### 2.2 Participants

Eighteen individuals (10F, 24.1 ± 3.0 years) participated in a larger study at the University of Colorado Boulder. To examine the mechanisms of potential hemoglobin mass changes after AIH, 12 individuals (6F, 24.2 ± 6.1 yrs) participated in a separate protocol including the measurement of serum EPO (additional subject characteristics can be found in Table 1). Participants were included if they were healthy and between the ages of 18–75 years old and excluded from the study if they had donated blood in the last 8 weeks, if they were smoking at the time of the study or had participated in tobacco activities up to 8 weeks prior to participation, or if they had a diagnosis of anemia that had not been resolved for at least 6 months.

**TABLE 1:** Participant characteristics. Data represent mean ± standard deviation. EPO–erythropoietin.

Characteristic	Non-EPO group (n = 18, 10F)	EPO group (n = 12, 6F)
Age (years)	24.1 ± 3.0	24.2 ± 6.1
Height (in)	66.3 ± 3.3	67.1 ± 2.8
Body Mass (kg)	68.7 ± 13.4	66.2 ± 10.9
Systolic blood pressure (mmHg)	123 ± 11.2	125 ± 14.3
Diastolic blood pressure (mmHg)	78.4 ± 10.0	76.3 ± 7.8

### 2.3 Acute intermittent hypoxia protocol

**FIGURE 1:**
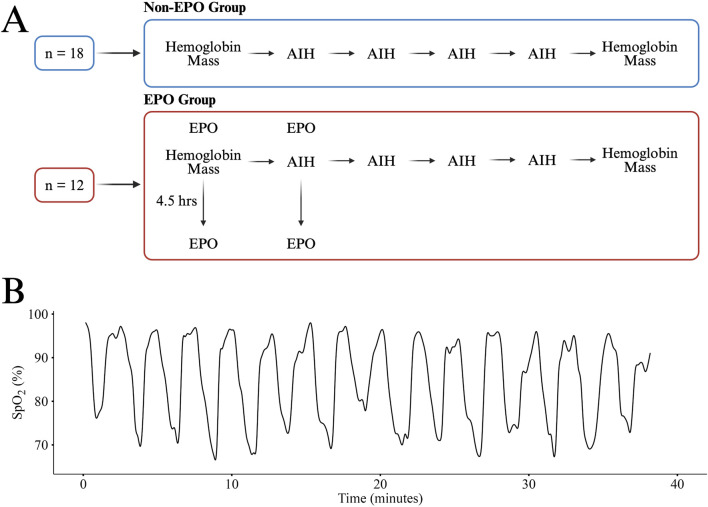
An overview the study design for the Non-EPO and EPO Groups **(A)**. A representative SpO_2_ curve from one participant in the EPO group during a single session of AIH **(B)**. All participants received the same AIH protocol and had similar responses. Panel A created in BioRender.

A single session of AIH consisted of fifteen 90 s intervals of breathing ∼9% O_2_ provided by a hypoxic generator (Hypoxico, NY, USA) (Tan et al., 2020b) with 60 s intervals of breathing room air (∼21% O_2_) in between. During each AIH dose, participants were monitored for pulse oximetry (SpO_2_) and heart rate at a frequency of 1 Hz and blood pressure was taken every five intervals (Masimo, CA, USA). The thresholds to pause AIH were defined as an SpO_2_ below 70%, a systolic blood pressure above 160 mmHg, or a heart rate above 160 bpm while AIH would resume once SpO_2_ returned to 80%, blood pressure was below 140 mmHg, or heart rate was below 140 bpm, respectively. Across all sessions, only SpO_2_ reductions required temporary pauses. All participants completed the 15 intervals without adverse events and no sessions required pausing due to elevated blood pressure or heart rate. In individuals for which we collected EPO (n = 12), SpO_2_ was recorded every 10 s over the course of each hypoxia session. An example of an SpO_2_ curve from a single AIH session is depicted in Figure 1.

### 2.4 Hemoglobin mass test

Total hemoglobin mass was measured via the optimized carbon monoxide rebreathing procedure (oCOR), as described previously (Schmidt and Prommer, 2005). Briefly, participants were asked to sit without moving for 10 min before the beginning of the procedure to allow fluid compartments to stabilize. Once participants had rested, a venous blood draw was taken for the measurement of hemoglobin concentration and hematocrit. Following the blood draw, the oCOR procedure consisted of rebreathing 99.5% CO (1.2 mL/kg for males and 1 mL/kg for females) along with 3 L of 100% O_2_ in a closed circuit for 2 minutes. Capillary blood samples were collected from the fingertip before and 5 minutes after the rebreathing procedure. In addition, end-tidal CO concentration was determined using a portable CO detector (Pac7000; Draeger, Luebeck, Germany) before and 2 minutes after the carbon monoxide rebreathing procedure. Hemoglobin concentration and percent carboxyhemoglobin (%CO) were determined in sextuplicate using an OSM3 hemoximeter (Radiometer, Copenhagen, Denmark). Hematocrit was measured in sextuplicate via microcentrifugation. During the rebreathing procedure, the circuit was monitored for CO leaks using the same portable detector as above (Ryan et al., 2011).

### 2.5 Serum erythropoietin and oxygen saturation

Approximately 3 mL of venous blood was collected into vacutainers containing serum separator and clot activator (Becton Dickinson, NJ, USA) and allowed to clot for 45 min. After clotting, tubes were centrifuged at ∼1600 x g for 15 min. Then, serum was aliquoted into six 1 mL cryogenic storage vials (Fisher Scientific, PA, USA) and stored for a minimum of 4 weeks at −70 °C before analysis. Serum EPO was measured with enzyme-linked immunosorbent assays (ELISA; R&D Systems, MN, USA and Abcam, Cambridge, UK). One plate was used to determine optimal dilution for our serum samples prior to EPO analysis and EPO samples were analyzed across 2 R&D plates and one Abcam plate. Each plate was run according to the manufacturer’s specifications. Serum EPO concentration was measured in duplicate. The intra-assay coefficients of variation for the three EPO plates were 2.39%, 2.33%, and 1.29%, respectively.

The mean SpO_2_ nadir of each desaturation was calculated by computing the average of the bottom 50 s of each desaturation within a given AIH session. SpO_2_ nadir during intermittent hypoxia has been reported previously (Knaupp et al., 1992). In this study, it will be used to compare to previous studies that either reported mean nadir or average SpO_2_ under hypoxia (Knaupp et al., 1992; Wojan et al., 2021; 2023).

### 2.6 Statistical analysis

Statistical analyses were performed in RStudio (v2024.12.1 + 563) with a significance level of α = 0.05. Data are presented as means ± standard deviation or confidence intervals. Normality and homoscedasticity were evaluated with Shapiro-Wilk tests and Levene’s tests, respectively. None of our data violated normality or homoscedasticity assumptions. Comparisons before and after AIH as well as the interaction effect of control or AIH day were compared using linear mixed model analyses, with subject as a random effect. Significance of fixed effects was evaluated using Satterthwaite approximations.

## 3 Results

### 3.1 Hemoglobin mass

**FIGURE 2:**
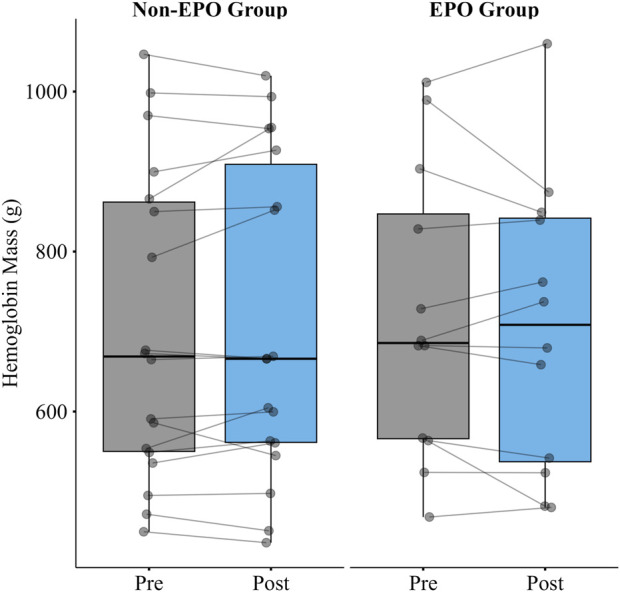
Hemoglobin mass pre and post 4 consecutive days of AIH in the Non-EPO Group (n = 18) and the EPO Group (n = 12). There was no significant increase in hemoglobin mass after 4 consecutive days of AIH in either group.

There was no significant increase in hemoglobin mass after four consecutive days of AIH in the first group with no serum EPO collection (n = 18; Δ −8.2g [-24.3, 8.0]; p = 0.30), or in the second group for which we collected EPO (n = 12; Δ 12.6g [-19.9,45.0]; p = 0.41) (Figure 2).

### 3.2 Serum EPO and oxygen saturation

**FIGURE 3:**
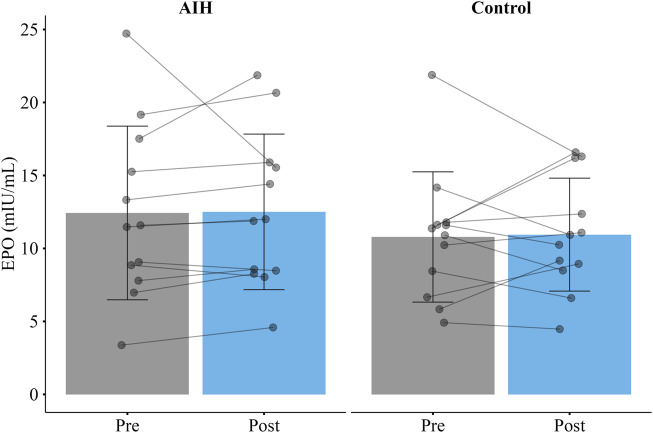
Mean erythropoietin (EPO) before and 4.5 h after a single session of acute intermittent hypoxia (AIH) or a control visit (Control) in the EPO group. There was no significant increase in EPO after AIH as compared to the control visit. Error bars represent standard deviation.

There was no significant increase in serum EPO after AIH as compared to control ( Δ EPO (AIH) = 0.07 mIU/mL [-1.95, 2.10]; Δ EPO (control) = 0.16 mIU/mL [-1.92, 2.24]; *p* = 0.96). In addition, mean baseline EPO on the AIH day was not significantly different from mean baseline EPO on the control day (12.43 mIU/mL [8.65, 16.21] vs. 10.78 mIU/mL [7.95, 13.62]; *p* = 0.15) (Figure 3). Mean SpO_2_ nadir of each desaturation for all AIH sessions was 73.16% ± 3.9%.

## 4 Discussion

The purpose of this study was to evaluate whether hemoglobin mass increased following four consecutive days of AIH. Accordingly, we found that four consecutive days of AIH did not increase hemoglobin mass in able-bodied individuals. In addition, we found that compared to control, AIH did not increase serum EPO, further emphasizing that this AIH protocol, when repeated across four consecutive days, is not sufficient to increase hemoglobin mass.

Although hemoglobin mass was not measured beyond the immediate study window, increases are unlikely in the absence of an EPO response. The present study aimed to evaluate whether hemoglobin mass could change within the same timeframe in which this AIH protocol has previously been associated with physiological improvements. Consecutive days of AIH combined with walking training improves walking endurance in individuals with iSCI compared to sham and walking training (Tan et al., 2021) and AIH improves the metabolic cost associated with walking during a motor learning task (Bogard et al., 2024). In the latter study, authors speculated that improvements in metabolic power may have been augmented by changes in endurance capacity and oxygen transport (Bogard et al., 2024). However, the current study suggests that walking improvements observed after four to five consecutive days of AIH do not appear to be explained by increases in hemoglobin mass.

To better understand the lack of increase in hemoglobin mass, we investigated the effect of this AIH protocol on serum EPO. No rise in serum EPO was observed, supporting the interpretation that erythropoiesis was not initiated. To our knowledge, this is the first study to evaluate EPO after hypoxia was delivered in repeated brief intervals at the present dose and duration. Previous work has shown that distinct AIH paradigms increase serum EPO after just one session of AIH (Knaupp et al., 1992; Wojan et al., 2021; 2023). In the study by Knaupp et al., participants were exposed to 4 hours of breathing 10.5% O_2_ for intervals of 2.5 min with 1.5 min of rest in between. Alternatively, Wojan et al.‘s group utilized eight 4-min intervals of breathing hypoxia to a targeted SpO_2_ of 80% separated by approximately 2 minutes of breathing normoxia (Wojan et al., 2021; 2023). Both the duration of hypoxic intervals and overall duration of AIH session were shorter in the present study than in the above studies. Induction of EPO mRNA at the cellular level increases exponentially as a function of time (Fandrey and Bunn, 1993). Therefore, the duration of each AIH session or hypoxic interval in our AIH protocol may simply have been too short to induce erythropoiesis, irrespective of repetition over consecutive days.

The average nadir during intermittent hypoxia in previous studies ranges from ∼75–82%, while the present study observed a lower average of 73% (Knaupp et al., 1992; Wojan et al., 2021; 2023). Similar to the relationship between EPO induction and time, there is a significant exponential increase in EPO mRNA expression in cells incubated with progressively decreased oxygen pressures (Fandrey and Bunn, 1993) and lower SpO_2_ is likely indicative of lower oxygen exposure at the tissues. Given the lower average SpO_2_ nadirs in this study, it is reasonable to believe that EPO would have increased. However, the physiological responses to hypoxia may be characterized by a balance between the magnitude of SpO_2_ decline and duration (Millet et al., 2016). Together with previous intermittent hypoxia studies, our data suggest that the greater level of desaturation in this study, as indicated by lower SpO_2_ nadir, was not sufficient to overcome the lower time under hypoxia.

In summary, four consecutive days of this AIH protocol does not increase hemoglobin mass in able-bodied individuals. Future research is needed to identify the features of intermittent protocols that are sufficient to induce erythropoiesis and should also examine other physiological pathways that may contribute to improved walking endurance in able-bodied individuals and those with functional limitations or health conditions.

## Data Availability

The raw data supporting the conclusions of this article will be made available by the authors, without undue reservation.
